# Instruments and consumables

**Published:** 2011-12

**Authors:** Ingrid Mason, Catherine Cross

**Affiliations:** CBM Medical Advisor, PO Box 58004, 00200 City Square, Ring Road Parklands, Nairobi, Kenya.; Formerly Manager, International Programmes, Sightsavers, Grosvenor Hall, Bolnore Road, Haywards Heath, West Sussex, RH16 4BX, United Kingdom.

**Figure:**
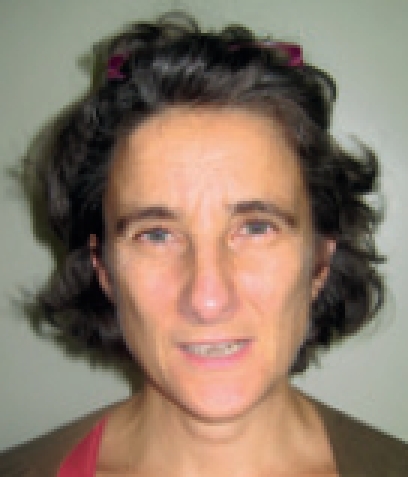
Ingrid Mason

**Figure:**
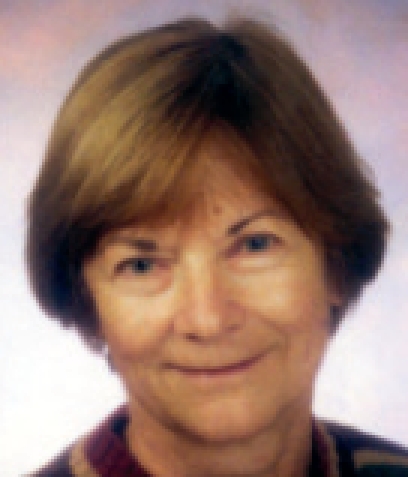
Catherine Cross

Our recent issue on “Equipment for eye care” (number 73, September 2010) addressed the importance of equipment in the delivery of eye care and covered maintenance, repair, training, purchasing, and donations. This issue (number 76) addresses similar concerns around surgical instruments and consumables.

For an eye unit to function, instruments must be carefully managed so that they remain in good repair and can be replaced quickly if needed. Consumables, such as stationery, spare parts, surgical supplies, and medicines, are fast-moving and must be managed so that they are always available and do not go out of date. To make this possible, systems must be in place that support scheduled maintenance and repair activities, monitoring of stock levels, and co-ordinated purchasing of instruments and consumables.

Without usable instruments and an adequate supply of consumables, not only does equipment stand idle, but so also does the eye care team — while patients wait for treatment or are turned away.

## Who is responsible?

The person in charge of an eye unit, usually the ophthalmologist or programme manager, is ultimately responsible to ensure that instruments and consumables are available when needed. Active oversight by the person in charge is vital, but in many eye units the day-to-day responsibility for purchasing and supplies management is delegated to another staff member, normally referred to as the stores manager.

**Figure:**
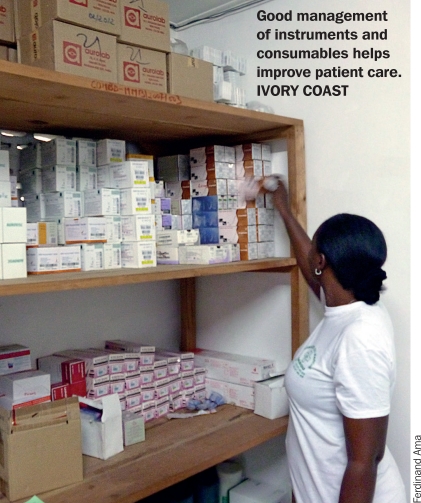


The stores manager is responsible for keeping an inventory of all consumables, finding and dealing with suppliers, planning orders, making purchases, receiving and unpacking deliveries, and physically arranging the stock in the stores area. Although the ordering and inventory management associated with instruments may also be dealt with by the stores manager, there may be a dedicated instruments person who will manage the necessary maintenance and repair protocols, including systems for reporting faulty instruments.

However, every member of the eye care team is responsible for instruments and consumables. Whatever your area of work, you must look after the items you use: identify low stocks or faulty items and communicate this to the stores manager, the instruments person, or the person in charge of your eye unit.

Whether you are an eye unit secretary who sees your supply of printed forms dwindling, or a nurse in a clinic where there are incomplete cataract sets, doing something about it is your responsibility — and we hope that this issue will show you how.

